# Increase in Meningococcal Serogroup W Disease, Victoria, Australia, 2013–2015

**DOI:** 10.3201/eid2210.151935

**Published:** 2016-10

**Authors:** Kylie S. Carville, Kerrie Stevens, Asma Sohail, Lucinda J. Franklin, Katherine A. Bond, Aicha Brahmi, Finn Romanes, Katherine S. Ong

**Affiliations:** Department of Health and Human Services, Melbourne, Victoria, Australia (K.S. Carville, A. Sohail, L.J. Franklin, A. Brahmi, F. Romanes, K.S. Ong);; Microbiological Diagnostic Unit Public Health Laboratory, Melbourne (K. Stevens, K.A. Bond);; Austin Health, Heidelberg, Victoria, Australia (K.A. Bond)

**Keywords:** Neisseria meningitidis, bacteria, meningitis, meningococcal serogroup W disease, invasive meningococcal disease, meningococcal infections, serogroup W, epidemiology, Victoria, Australia

## Abstract

In Victoria, Australia, invasive meningococcal disease caused by *Neisseria meningitidis* serogroup W increased from 4% of all cases in 2013 to 30% in 2015. This increase resulted largely from strains similar to those in the serogroup W sequence type 11 clonal complex, previously described in the United Kingdom and South America.

*Neisseria meningitidis* serogroup W (MenW) disease was believed to have little epidemic potential before the first international outbreak in 2000, which was related to the Hajj, and has subsequently been described in the African meningitis belt, South Africa, and Saudi Arabia (*1*). This serogroup emerged in the South Cone of South America in the mid-2000s and in the United Kingdom in 2009–2010. Strains belonging to sequence type (ST) 11 are the most prevalent; those from the South Cone of South America are part of ST11 but have diversified (*1**,**2*).

Since 2009–2010, the United Kingdom (England and Wales) has seen an increase in disease caused by 1 endemic hypervirulent strain of MenW that began in older adults and subsequently spread to persons in all age groups (*3**,**4*). Historically, MenW accounted for <5% of laboratory-confirmed cases of invasive meningococcal disease (IMD) in England, but in 2014–2015, this serogroup was responsible for 25% of all cases (*3*). The ST11 strain was shown by whole-genome sequencing (WGS) to be similar to strains in the South Cone nations of South America (*2**,**4**,**5*). In response, vaccination programs against MenW have been initiated in the United Kingdom and Chile (*1**,**3*).

The Department of Health and Human Services (Melbourne, Victoria, Australia) collects information from doctors and laboratories about diagnoses of certain health-related conditions in Victoria under the Public Health and Wellbeing Act, 2008. The incidence of IMD in Victoria decreased from 2.5 cases/100,000 population (125 cases) in 2003, when the conjugate meningococcal C vaccine was added to the National Immunization Program, to 0.6 cases/100,000 population in 2014 (*6*).

Serogroup B is currently the most common cause of IMD in Victoria. However, the department has observed a significant increase in reports of MenW; 17 (30% of IMD cases) cases were reported in 2015, compared with 4 (12%) cases in 2014 and 1 (4%) case in 2013 (p<0.01 by test of proportions for 2013–2015) (Figure 1). In 2015, a total of 55 confirmed cases of IMD were reported to the department: 29 were caused by serogroup B, 17 serogroup W, 9 serogroup Y, and 1 probable case without laboratory confirmation. We analyzed the status of meningococcal serogroup W disease in Victoria during 2013–2015.

**Figure 1:**
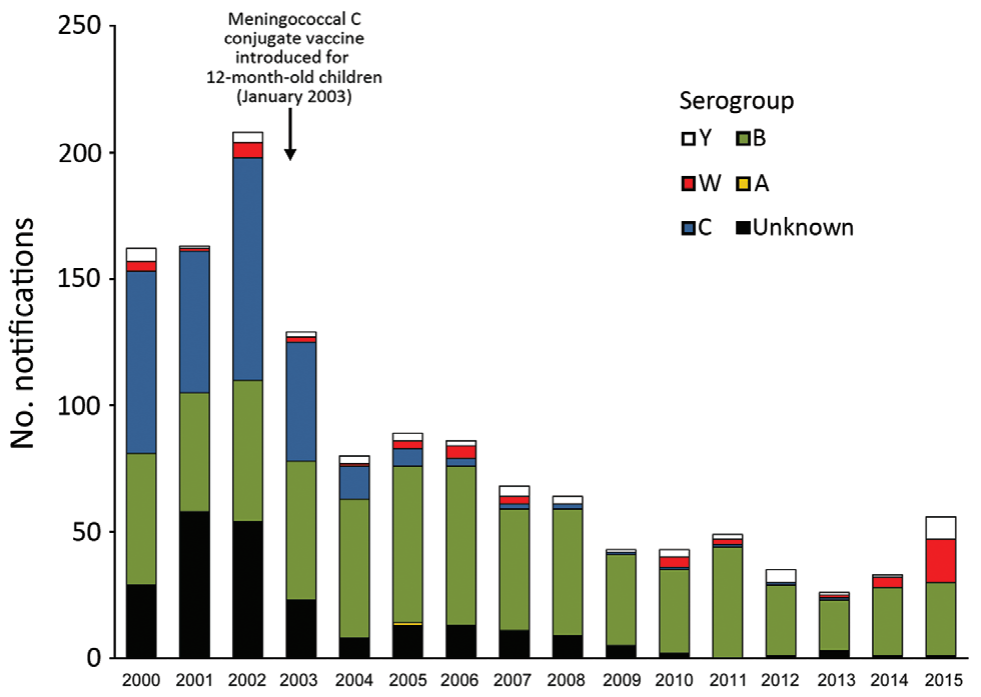
Invasive meningococcal disease notifications, by serogroup and year, Victoria, Australia, January 1, 2000–December 31, 2015.

## The Study

The 22 MenW cases reported during January 1, 2013–December 31, 2015, were in older persons than is typically seen for IMD (9 cases were in persons >70 years of age). Median age of these 22 case-patients was 56 years; median age was 19 years for patients with serogroup B infections. Eleven (50%) MenW cases were in women.

Bacteremia was the predominant manifestation among MenW case-patients (55%), followed by septic arthritis (with or without bacteremia) (18%) (Table). Epiglottitis and pneumonia were also observed. Seven (32%) MenW case-patients were admitted to intensive care units. The case-fatality rate was low (5%); 1 death occurred in a young man who had no previous illnesses. In comparison, the most common manifestation in patients with serogroup B disease during the same period was meningitis (45%), followed by bacteremia (37%), and meningitis with bacteremia (17%). The case-fatality rate for patients with serogroup B disease was also low (1.3%).

**Table:** Characteristics of 22 patient with invasive meningococcal serogroup W disease, Victoria, Australia, January 1, 2013–December 31, 2015*

Characteristic	All serogroup W	W:P1.5,2:F1-1:ST11†	Other ST†
Median age (range), IQR, y	56 y (<1 mo–89 y), 23–72 y	56 y (<1 mo–85 y), 25–72 y	45 y (<1 mo–89 y), 1–84 y
Indigenous status			
Aboriginal or Torres Strait Islander	0	0	0
Non-Aboriginal	21 (95)	16 (100)	5 (100)
Unknown	1 (5)	0	0
Manifestation with petechial rash	2 (10)	1 (6)	0
Manifestation			
Bacteremia	12 (55)	8 (50)	3 (60)
Septic arthritis	4 (18)	3 (19)	1 (20)
Meningitis	1 (5)	0	1 (20)
Meningitis and bacteremia	2 (9)	2 (12)	0
Pneumonia	2 (9)	2 (12)	0
Epiglotittis	1 (5)	1 (6)	0
Admission to intensive care unit	7 (32)	6 (38)	0
Death	1 (5)	0	0
Total	22	16	5

*Values are no. (%) unless otherwise indicated. IQR, interquartile range; ST, sequence type. †Excludes 1 patient for whom ST was not determined.

Variable region typing (finetyping) for *PorA* and *FetA* genes was conducted for 22 specimens from MenW case-patients (21 isolates and 1 cerebrospinal fluid sample). WGS was conducted for 21 isolates, and multilocus sequence typing was conducted in silico (*7*). One case, in the person who died, was diagnosed by PCR of a cerebrospinal fluid sample (WGS was not possible for this patient).

**Figure 2:**
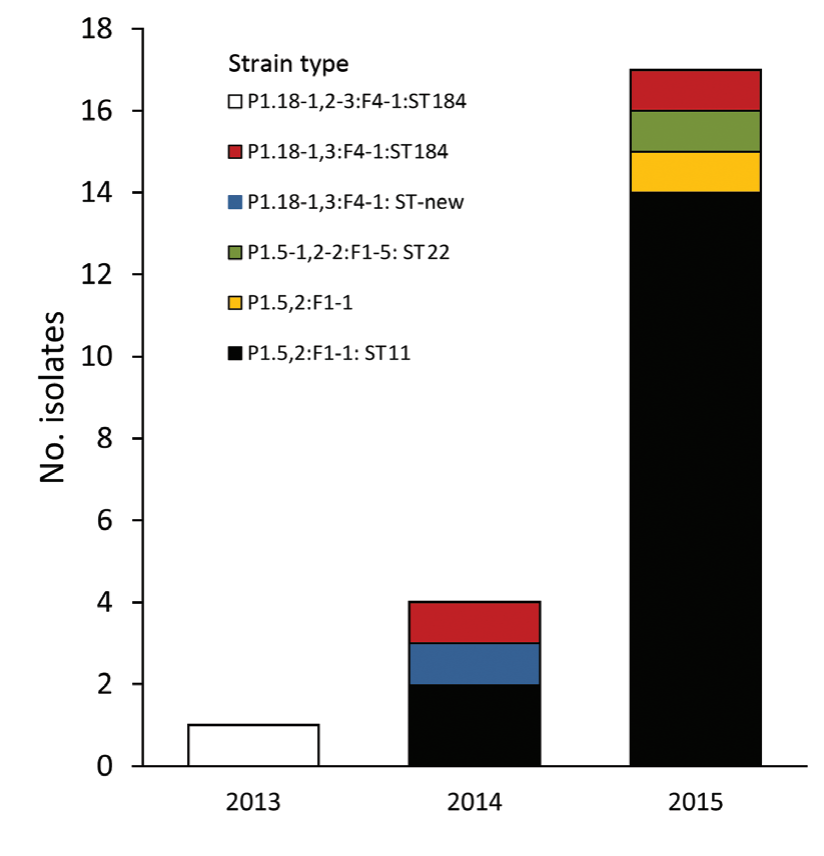
Finetypes of 17 invasive meningococcal serogroup W isolates, Victoria, Australia, January 1, 2000–December 31, 2015.

Seventeen specimens were identified as strain type W:P1.5,2:F1–1; sixteen were ST11 (Table). This strain type has been observed only once in Victoria for an isolate in 2006 (Microbiological Diagnostic Unit Public Health Laboratory, unpub. data). This pattern is similar to that for the predominant strain type that emerged in the United Kingdom (*4*). The remaining 5 isolates were not ST11. Two isolates had the same strain type (P1.18-1,3:F4-1:ST184), and the remaining strain types were observed only once each (Figure 2). Phylogenetic analysis showed that isolates from Victoria clustered with isolates from the United Kingdom and South America but not with isolates from South Africa or Hajj strains (*8*).

Among the 22 case-patients, 3 did not have any close contacts (i.e., persons who lived in the same household or with whom they had intimate physical contact). Risk factors were not common. Five smokers were identified, and 1 person was immunosuppressed (early myeloma). No case-patients had traveled during the 1–2 weeks before onset of illness. Additional travel history was obtained for 15 of 16 case-patients infected with ST11. Travel among close contacts was reported in the 12 months preceding onset of illness in 4 instances to countries in Southeast Asia, southeastern Europe, and the United Kingdom.

## Conclusions

Routine surveillance detected an increase in invasive MenW disease in Victoria, Australia. Although most disease manifestations were mild, there was 1 death in an otherwise healthy young adult. Milder manifestations might be related to older ages of many case-patients or be influenced by serogroup. Overrepresentation of MenW (and serotype Y) in patients with septic arthritis, pericarditis, and pneumonia has been described, often in older persons, but meningococcal pneumonia might be more common among younger persons infected with these serogroups (*9**–**11*). Other states and territories in Australia have not shown a similar increase in MenW disease.

Absence of travel for most case-patients indicates that transmission of W:P1.5,2:F1-1:ST11 might be endemic in Victoria, although investigations remain ongoing. There is substantial population movement between Australia and the United Kingdom; the United Kingdom is the primary source of permanent migrants (*12*) and a major source and destination for short-term visits (*13*). This population movement and genetic similarity suggest that the strain was originally introduced from the United Kingdom.

The atypical nature of disease manifestations observed could result in underdetection of cases. Absence of close contacts for some case-patients raises questions about disease transmission, including potential for transmission from persons not typically defined as being close contacts (e.g., a carrier to which exposure is short). ST11 strains have been described as being highly transmissible and persistent (including carriage for several months postacquisition) (*14**,**15*).

In response to the increase in MenW cases, the Department of Health and Human Services advised health professionals in Victoria to consider MenW disease in the differential diagnosis of atypical infections in older patients. Enhanced surveillance has been initiated to capture more detail on travel history and contacts to better understand the current epidemiology of MenW. WGS, which was not used previously for detection of IMD, is being used in conjunction with standard typing methods for higher resolution of genetic relatedness of isolates. Although no changes have been made in vaccination recommendations for Victoria, additional data will provide useful information for future discussions regarding merits of vaccination programs.

The response in Victoria benefited from documentation of rapid endemic expansion of similar strains of MenW in the United Kingdom and South America. Public health officials in Victoria were able to detect and monitor the situation early, and the public health response was aided by actions in these 2 other regions. Given that MenW ST11 was established in older populations in the United Kingdom before spreading to younger populations and has been described as being hypervirulent (*3*), disease progression in Victoria is being closely monitored for a similar transition. The situation is evolving, epidemiologic and molecular investigations are ongoing, and other jurisdictions are encouraged to monitor emergence of this strain.

## References

[1] Abad R, López EL, Debbag R, Vázquez JA. Serogroup W meningococcal disease: global spread and current affect on the Southern Cone in Latin America. Epidemiol Infect. 2014;142:2461–70. 10.1017/S0950268814001149 24831052 PMC9151320

[2] Araya P, Fernández J, Del Canto F, Seoane M, Ibarz-Pavón AB, Barra G, *Neisseria meningitidis* ST-11 clonal complex, Chile 2012. Emerg Infect Dis. 2015;21:339–41. 10.3201/eid2102.140746 25625322 PMC4313638

[3] Campbell H, Saliba V, Borrow R, Ramsay M, Ladhani SN. Targeted vaccination of teenagers following continued rapid endemic expansion of a single meningococcal group W clone (sequence type 11 clonal complex), United Kingdom 2015. Euro Surveill. 2015;20:21188. 10.2807/1560-7917.ES2015.20.28.21188 26212140

[4] Ladhani SN, Beebeejaun K, Lucidarme J, Campbell H, Gray S, Kaczmarski E, Increase in endemic *Neisseria meningitidis* capsular group W sequence type 11 complex associated with severe invasive disease in England and Wales. Clin Infect Dis. 2015;60:578–85. 10.1093/cid/ciu881 25389259

[5] Lucidarme J, Hill DM, Bratcher HB, Gray SJ, du Plessis M, Tsang RS, Genomic resolution of an aggressive, widespread, diverse and expanding meningococcal serogroup B, C and W lineage. J Infect. 2015;71:544–52. 10.1016/j.jinf.2015.07.007 26226598 PMC4635312

[6] Australian Government Department of Health. National notifiable diseases surveillance system [cited 2016 Mar 2]. http://www9.health.gov.au/cda/source/cda-index.cfm

[7] Jolley KA, Brehony C, Maiden MC. Molecular typing of meningococci: recommendations for target choice and nomenclature. FEMS Microbiol Rev. 2007;31:89–96. 10.1111/j.1574-6976.2006.00057.x 17168996

[8] Bond KA, Stevens K, Bulach D, Carville K, Ong KS, Howden BP. Rising incidence of invasive meningococcal disease caused by Neisseria meningitidis serogroup W in Victoria. Med J Aust. 2016;204:265–6. 10.5694/mja15.01222 27078598

[9] Koppes GM, Ellenbogen C, Gebhart RJ. Group Y meningococcal disease in United States Air Force recruits. Am J Med. 1977;62:661–6. 10.1016/0002-9343(77)90867-1 404877

[10] Vienne P, Ducos-Galand M, Guiyoule A, Pires R, Giorgini D, Taha MK, The role of particular strains of *Neisseria meningitidis* in meningococcal arthritis, pericarditis, and pneumonia. Clin Infect Dis. 2003;37:1639–42. 10.1086/379719 14689345

[11] Winstead JM, McKinsey DS, Tasker S, De Groote MA, Baddour LM. Meningococcal pneumonia: characterization and review of cases seen over the past 25 years. Clin Infect Dis. 2000;30:87–94. 10.1086/313617 10619738

[12] Australian Bureau of Statistics. 3412.0 Migration, Australia, 2013–14. 2015 [cited 2015 Oct 27]. http://www.abs.gov.au/ausstats/abs@.nsf/PrimaryMainFeatures/3412.0?OpenDocument

[13] Australian Bureau of Statistics. 3401.0 Overseas arrivals and departures, Australia, June 2015 [cited 2015 Oct 27]. http://www.abs.gov.au/ausstats/abs@.nsf/products/961B6B53B87C130ACA2574030010BD05

[14] Nicolas P, Ait M’barek N, Al-Awaidy S, Al Busaidy S, Sulaiman N, Issa M, Pharyngeal carriage of serogroup W135 *Neisseria meningitidis* in Hajjees and their family contacts in Morocco, Oman and Sudan. APMIS. 2005;113:182–6. 10.1111/j.1600-0463.2005.apm1130305.x 15799761

[15] Wilder-Smith A, Barkham TM, Ravindran S, Earnest A, Paton NI. Persistence of W135 *Neisseria meningitidis* carriage in returning Hajj pilgrims: risk for early and late transmission to household contacts. Emerg Infect Dis. 2003;9:123–6. 10.3201/eid0901.020131 12533295 PMC2873737

